# Heat Shock Alters the Expression of Schizophrenia and Autism Candidate Genes in an Induced Pluripotent Stem Cell Model of the Human Telencephalon

**DOI:** 10.1371/journal.pone.0094968

**Published:** 2014-04-15

**Authors:** Mingyan Lin, Dejian Zhao, Anastasia Hrabovsky, Erika Pedrosa, Deyou Zheng, Herbert M. Lachman

**Affiliations:** 1 Department of Genetics, Albert Einstein College of Medicine, Bronx, New York, United States of America; 2 Department of Neurology, Albert Einstein College of Medicine, Bronx, New York, United States of America; 3 Department of Psychiatry and Behavioral Sciences, Albert Einstein College of Medicine, Bronx, New York, United States of America; 4 Dominick Purpura Department of Neuroscience, Albert Einstein College of Medicine, Bronx, New York, United States of America; 5 Department of Medicine, Albert Einstein College of Medicine, Bronx, New York, United States of America; University of South Florida, United States of America

## Abstract

Schizophrenia (SZ) and autism spectrum disorders (ASD) are highly heritable neuropsychiatric disorders, although environmental factors, such as maternal immune activation (MIA), play a role as well. Cytokines mediate the effects of MIA on neurogenesis and behavior in animal models. However, MIA stimulators can also induce a febrile reaction, which could have independent effects on neurogenesis through heat shock (HS)-regulated cellular stress pathways. However, this has not been well-studied. To help understand the role of fever in MIA, we used a recently described model of human brain development in which induced pluripotent stem cells (iPSCs) differentiate into 3-dimensional neuronal aggregates that resemble a first trimester telencephalon. RNA-seq was carried out on aggregates that were heat shocked at 39°C for 24 hours, along with their control partners maintained at 37°C. 186 genes showed significant differences in expression following HS (p<0.05), including known HS-inducible genes, as expected, as well as those coding for *NGFR* and a number of SZ and ASD candidates, including *SMARCA2, DPP10, ARNT2, AHI1* and *ZNF804A.* The degree to which the expression of these genes decrease or increase during HS is similar to that found in copy loss and copy gain copy number variants (CNVs), although the effects of HS are likely to be transient. The dramatic effect on the expression of some SZ and ASD genes places HS, and perhaps other cellular stressors, into a common conceptual framework with disease-causing genetic variants. The findings also suggest that some candidate genes that are assumed to have a relatively limited impact on SZ and ASD pathogenesis based on a small number of positive genetic findings, such as *SMARCA2* and *ARNT2*, may in fact have a much more substantial role in these disorders - as targets of common environmental stressors.

## Introduction

Schizophrenia (SZ) and autism spectrum disorders (ASD) are among the most heritable neuropsychiatric disorders. An ever-expanding number of susceptibility genes is being identified through genome wide association studies (GWAS), copy number variant (CNV) analysis, and exome sequencing [1]–[15]. However, environmental factors also appear to play a role, especially maternal immune activation (MIA), which may be brought on by exposure to infectious diseases or autoimmune phenomena [16]–[30]. Supporting an infectious disease and/or autoimmune etiology in a subset of SZ patients is the association that has been found to markers in the HLA locus – one of the most robust GWAS findings in SZ genetics research (although non-immune effects of HLA antigens on brain development and neuronal function is a possible explanation for the association) [1], [14], [31]–[33].

The effects of MIA are mediated by a balance between proinflammatory and anti-inflammatory cytokines, such as interleukins 1β, 6, 10 and 13 [27], [34]–[36]. These cytokines are especially interesting in the context of neuropsychiatric disorders because they have well-established effects on neurogenesis and brain development, which could influence behavior in adult offspring. One model that is commonly used to test the effects of MIA is to treat pregnant animals with cytokines or agents that mimic exposure to infectious organisms, such as bacterial endotoxin (lipopolysaccharide; LPS), and polyinosinic: polycytidylic acid (poly I:C), after which the effects on behavior and neuronal function in the offspring are analyzed. For example, when pregnant mice are treated with poly I:C, altered prefrontal GABAergic gene expression occurs in their adult progeny [37]. Similarly, MIA in first trimester rhesus monkeys leads to offspring with increased repetitive behaviors and abnormal social interactions [38]. Prenatal exposure to low concentrations of poly I:C that do not lead to maternal symptoms or fetal death has also been found to cause impaired non-spatial memory and learning tasks in adult offspring, and decreased hippocampal reelin expression [39]. Similarly, prenatal exposure to poly I:C was shown to reduce the density of parvalbumin GABAergic interneurons in the CA1 region of the hippocampus, similar to that seen in the brains of patients with SZ [40]–[44]. Prenatal exposure to LPS has similar effects on brain development and long-term behavioral effects on offspring [42]–[47].

Although most studies examining the effect of MIA revolve around the effect of cytokines on neurogenesis and brain development, there are very few studies that have examined the effect of fever per se. One study that supports an effect is the finding that the risk of ASD in children born to mothers who experienced a febrile episode during pregnancy is attenuated by antipyretic medications [17]. In animal studies, prenatal exposure to LPS that was accompanied by a febrile reaction resulted in offspring with altered intrinsic excitability of CA1 pyramidal neurons [48]. In a large epidemiological study in Denmark, maternal influenza and febrile episodes were found to increase the risk of ASD, although the findings were not significant after multiple testing corrections were applied [16]. Interestingly, and perhaps somewhat paradoxically, there is also some suggestion that children with ASD have a transient improvement in symptoms following a febrile episode [49], [50]. Thus, there have been some interesting observations related to fever in SZ and ASD, but its potential role as a risk factor has not been well-studied.

As a first step towards understanding the potential effect of fever on the developing human brain, we are using a unique culture system developed by Mariani et al. in which iPSCs are manipulated with rostral neuralizing factors to produce 3-dimensional neuronal aggregates that model the developing first trimester telencephalon [51]. A modification of this culture system has recently been used to grow cortical structures and to model microcephaly [52]. We exposed 50 day old aggregates to HS (39°C for 24 hours) and analyzed transcripts genome-wide using RNA-seq. As expected, the expression level of a number of heat shock (HS) genes markedly increased. Interestingly, the expression of *NGFR* (nerve growth factor receptor) and several genes that have been implicated in the development of SZ, bipolar disorder (BD) and ASD were differentially expressed following HS.

## Methods

### Subjects and development of iPSCs from skin fibroblasts

All work involving iPSCs was approved by the Albert Einstein College of Medicine committee on clinical investigation. Participants in this study signed consent forms approved by The Albert Einstein College of Medicine Institution Review Board (IRB). Subjects were also recruited at the NIMH, Child Psychiatry Branch as part of an ongoing study on childhood onset schizophrenia directed by Dr. Judith Rapoport. Subjects in that study signed consents approved by the NIMH IRB. Consent was obtained by skilled members of the research teams who had received prior human subjects training. All lines used in this study were derived from healthy subjects who are serving as controls in ongoing studies in which iPSCs are being developed from patients with SZ who have 22q11.2 deletions. RNA-seq studies were carried out on 2 lines, which we designated as control 1 (C1) and control 2 (C2). C1 is an18 year old male and C2 is a 31 year old female. iPSC lines were generated from skin fibroblasts. In addition, we validated several genes of interest by quantitative real time PCR (qPCR; see below) using another control subject designated as C3, a 27 year old male. The reprogramming procedure is described in the Supporting Information file (Text S1).

### Neuronal Differentiation

RNA-seq was carried out on neuronal aggregates as described by Mariani et al. with slight modification (see Text S1) [51]. For the HS experiment, a group of 49 day old aggregates was placed in an incubator set at 39°C for 24 hours, while control sets of aggregates were maintained at 37°C. The incubator conditions were otherwise unchanged (ambient O_2_, 5% CO_2_, 85% humidity). After detaching the aggregates, total cellular RNA was isolated using the miRNeasy Kit (Qiagen) according to the manufacturer' protocol. An additional DNAse1 digestion step was performed to ensure that the samples were not contaminated with genomic DNA.

### RNA-seq

RNA was extracted from control and HS samples from day 50 mini-brain aggregates derived from the two iPSC lines (C1 and C2; HS1 and HS2). Paired end RNA-seq was carried on an Illumina HiSeq 2000. We obtained 101-bp mate-paired reads from DNA fragments of with an average size of 250-bp (standard deviation for the distribution of inner distances between mate pairs is approximately 100 bp). RNA-seq reads were aligned to the human genome (GRCh37/hg19) using the software TopHat (version 2.0.8) [53]. We counted the number of fragments mapped to each gene annotated in the GENCODE database (version 18) [54]. The category of transcripts is described at http://vega.sanger.ac.uk/info/about/gene_and_transcript_types.html. Transcript abundances were measured in Transcripts Per Million (TPM), which is calculated by multiplying the estimated fraction of transcripts made up by a given gene by 10^6^ [55]. The measure is independent of the mean expressed transcript length and is thus more comparable across samples, so it is favored over another popular transcript measure, FPKM [55]. We used DESeq (an R package developed by Anders and Huber) to evaluate differential expression from the count data [56]. Specifically, DESeq models the variance in fragment counts across replicates using the negative binomial distribution and tests whether, for a given gene, the change in expression strength between the two experimental conditions is significantly large as compared to the variation within each replicate group. In the end, only genes with average TPMs greater than 1 across samples were considered for differential expression. RNA-seq data have been deposited at the Gene Expression Omnibus (GEO) repository (accession # GSE53667).

### Quantitative real-time PCR (qPCR)

qPCR was carried out on reverse transcribed PCR. A detailed description and the primers used for this analysis can be found in Text S1.

## Results

**Figure 1:**
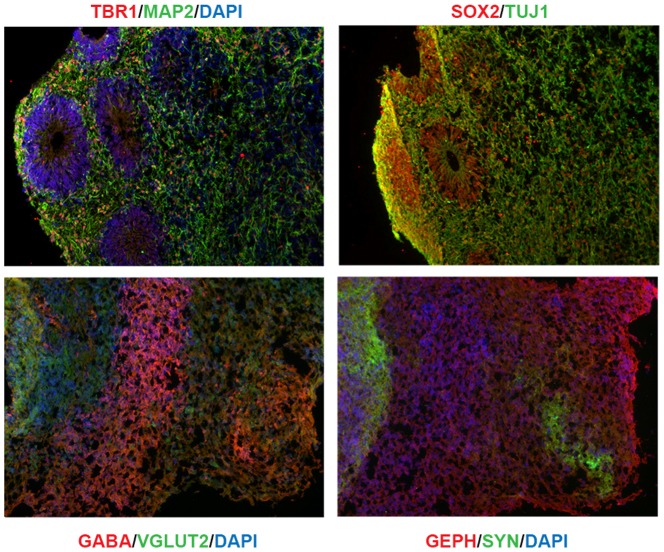
Neuronal aggregates, day 50. **Top panels: SOX2+ structures containing radial glial cells with surrounding field of neurons (MAP+, TUJ1+ cells).** Bottom left panel: Neurons contain layers of predominantly GABAergic and glutamatergic neurons (GABA+ and VGLUT2+, respectively). Bottom right panel: The neurons express pre and post synaptic proteins (synaptophysin/SYN, gephyrin/GEPH, respectively).

Neuronal aggregates were prepared from two control subjects (C1 and C2); an 18 year old male and 31 year old female, respectively. A representative aggregate is shown in Figure 1. Aggregates are composed of SOX2 positive, radial glial-containing structures surrounded by a field of neurons that are primarily GABAergic and glutamatergic. As seen in Figure 1, the aggregates express pre and postsynaptic markers (synaptophysin and gephryin, respectively). At day 49, while the control aggregates were maintained continuously at 37°C, another set of aggregates from each subject was exposed to HS (39°C for 24 hours; designated HS1 and HS2). RNA was then extracted from the control and HS samples on day 50, and subjected to RNA-seq. The number of RNA-seq reads and fraction of reads that mapped to the genome was similar for all samples (Table S1). In addition, the coefficient of variance was low (0.11 for the two controls; 0.19 for the two HS samples: Pearson correlation coefficients were 0.98 and 0.96, respectively), indicating high reproducibility of our RNA-seq data. The RNA-seq data show that the forebrain transcription factors, *FOXP2, GLI2, LHX1, LHX2, POU3F2* and *EMX2* are expressed, but not the hindbrain transcription factors *HOXA1,HOXA2, HOXB1, HOXB2,* and *HOXB3* (Table S2). The aggregates express a fairly heterogeneous mix of neurotransmitter receptor genes, although GABAergic and glutamatergic receptors predominate; the glutamate transporter genes, *SLC17A6* and *SLC17A7*, and the GABAergic transporter gene *SLC6A1* are also expressed. The aggregates express only a few serotonin and dopamine receptor subtype genes (*HTRA1, HTRA2* and *HTR5A*; *DRD2* and *DRD4*), and several nicotinic cholinergic receptor subtypes (*CHRNB1, CHRNA1, CHRN*A4, *CHRN*A7, *CHRNB2*, *CHRNB3* and *CHRNB4)*. The dopamine transporter (*SLC6A3*), serotonin transporter (*SLC6A4*) and cholinergic transporter (*SLC18A3*) genes are not expressed, although TH (tyrosine hydroxylase), a dopaminergic marker is. There were no significant differences in expression of any neurotransmitter receptor or transporter gene in response to HS, with the exception of *SLC17A7*, as described below.

**Figure 2:**
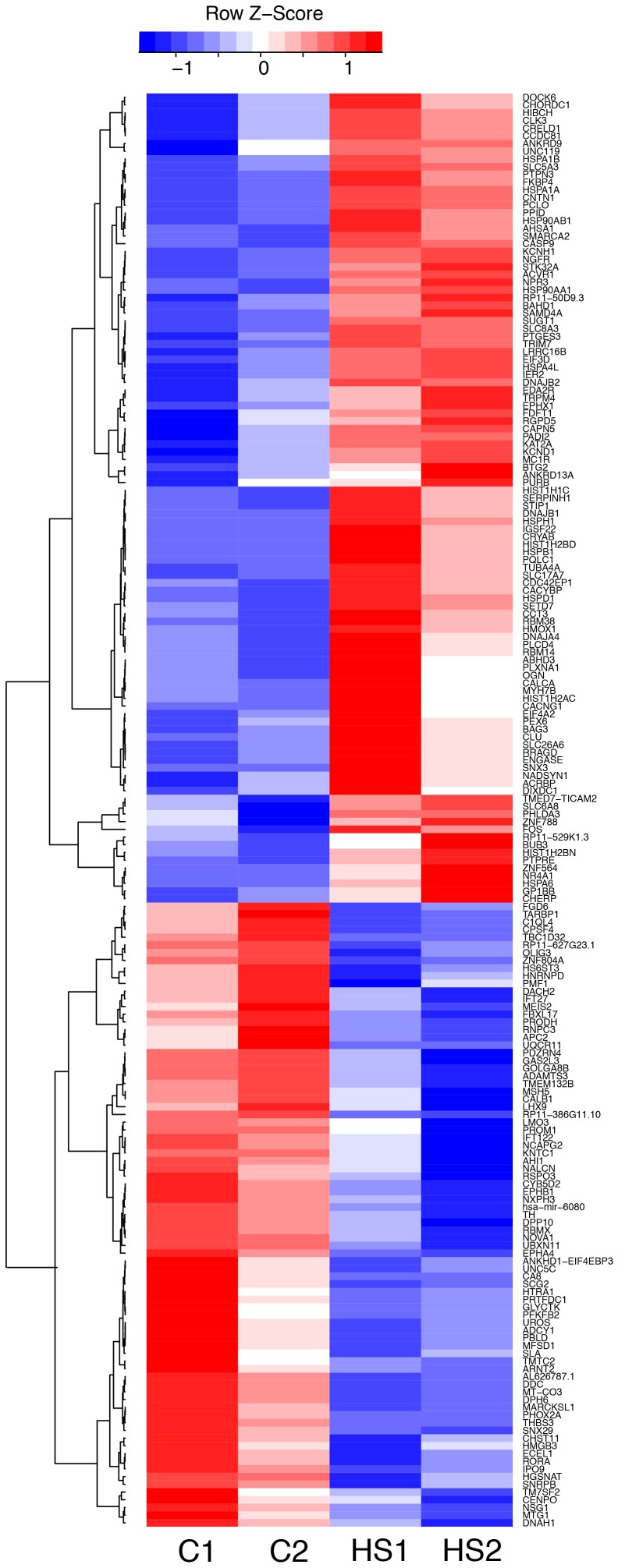
Heat map showing relative expression of 186 genes that exhibited significant change in gene expression following heat shock at a nominally significant level (p<0.05: 105 increased in expression; 81 decreased).

The expression level of 186 genes showed a nominally significant difference in expression following HS (p<0.05: 105 increased; 81 decreased), of which 12 achieved genome wide significance (q<0.05: all increased with HS) (Figure 2; Table S2). Among the 12 genes that increased most dramatically were 8 members of the HS gene family (*HSPA1A, HSPA1B, HSP90AA1, HSP90AB1, HSPH1, HSPA6, HSPA4L, DNAJB1*), and the HS protein chaperones *CRYAB* and *FKBP4*. In addition, several genes of interest with respect to neurogenesis and neuronal function were among the most HS-inducible genes, including *SLC5A3* (sodium/myo-inositol co-transporter), which regulates brain inositol levels, and *NGFR*, which codes for the nerve growth factor (NGF) receptor.

Among the most down-regulated genes were several that code for proteins involved in dopamine transmission, including *DDC* (dopamine decarboxylase), and *PHOX2A* (paired mesoderm homeobox protein 2A), a transcription factor that regulates *TH* and *DBH* (dopamine beta hydroxylase) gene expression [57]. In addition, other dopaminergic genes - *DBH*, *TH*, and *ADCY1* - were also down-regulated by HS. This is consistent with the finding that heat stress reduces TH immunoreactivity in striatal dopamine neurons in mice and impairs nigrostriatal dopaminergic neurons and motor function [58].

To identify which of the 186 genes could be the direct transcriptional targets of HS, we reanalyzed the ChIP-seq data that was collected by the ENCODE consortium for HSF1 in the HepG2 cell line [54], [59], since no ChIP-seq data for HSFs are available for human brains or neurons. We found that 28 of our 186 DE genes had at least one HSF1 ChIP-seq peak within 50 kb (P = 3.3e-5, hypergeometric test) (24 up-regulated by HS: *ABHD3, AHSA1, CACYBP, CCT3, CHORDC1, CLU, DNAJB1, DNAJB2, FKBP4, HIST1H2BN, HSP90AA1, HSP90AB1, HSPA1A, HSPA1B, HSPA6, HSPD1, HSPH1, KAT2A, PTGES3, SLC5A3, STIP1, SUGT1, TH, TUBA4A*; 4 down-regulated: *KNTC1, MSH5, SNX29, hsa-mir-6080*).

qPCR was used to validate some differentially expressed genes. Because of a lack of RNA remaining after RNA-seq, we were only able to validate a small number of genes. A significant 4-fold increase in *HSP90AB1* (Student's t-test, P = 0.006) and a 1.8-fold decrease in *ZNF804A* were found (P = 0.005), which confirmed the RNA-seq findings.

**Figure 3:**
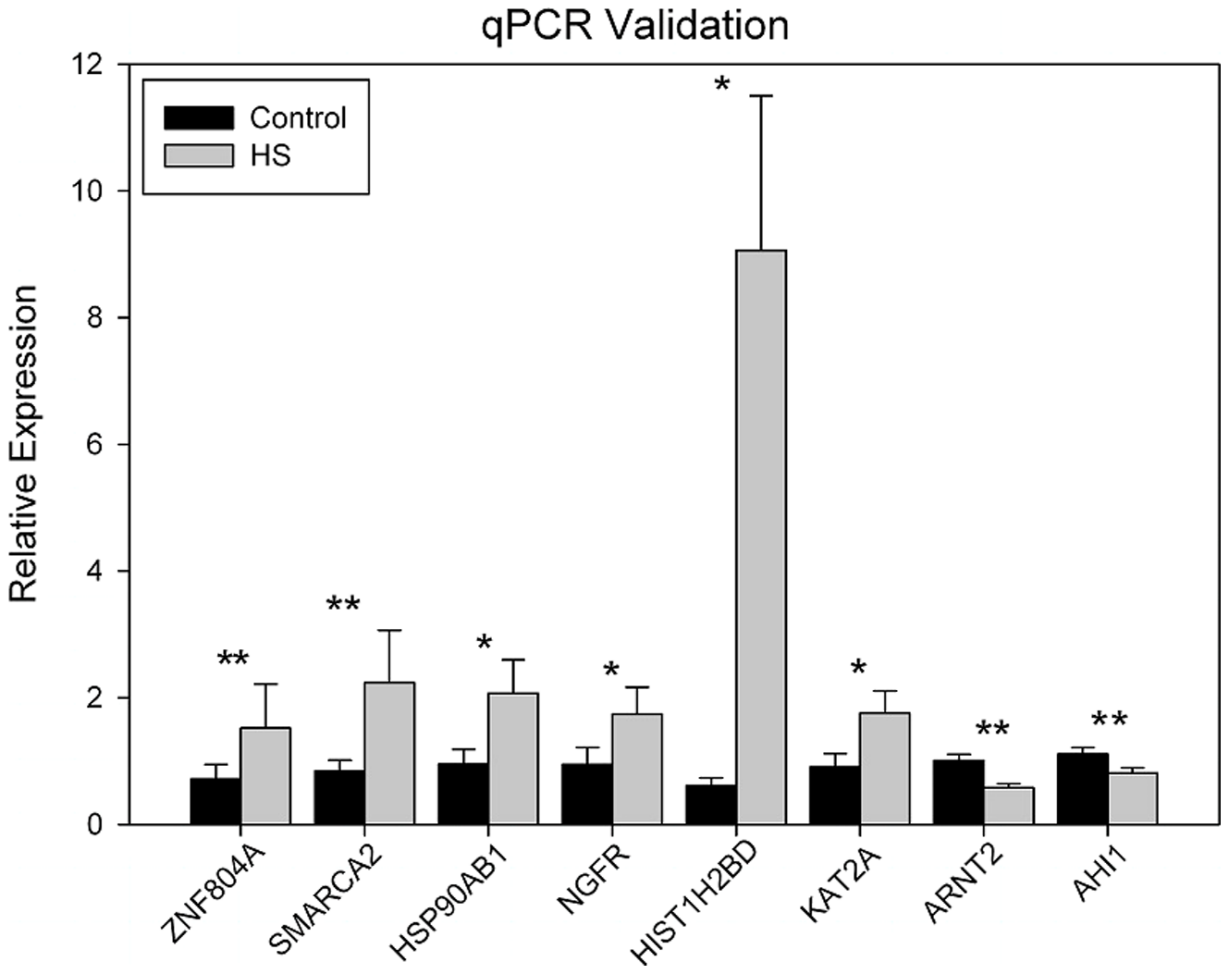
qPCR validation for C3 and HS3. Between 4–8 aggregates were analyzed individually in triplicate using the 2^−ΔΔCt^ relative expression method with β2-microglobulin (β2M) as a control gene. Each sample was normalized against a common control RNA. Mean values +/− standard deviation are shown and a student's t-test was performed. A single asterisk indicates a p<0.05; two asterisks indicate a p<0.01. The actual p-values are *ZNF804A* (0.0002), *SMARCA2* (0.0001), *HSP90AB1* (0.03), *NGFR* (0.04), *HIST1H2BD* (0.02), *KAT2A* (0.05), *ARNT2* (0.002), *AHI1* (0.006).

For further qPCR validation, we prepared multiple aggregates from a third control sample and carried out another HS experiment (C3 and HS3). As seen in Figure 3, significant differences in gene expression following HS were observed for every gene analyzed, which all changed in the same direction as in the RNA-seq findings, with the exception of *ZNF804A*, which increased in C3 upon HS, but decreased in C1 and C2.

### Pathway Analysis

All differentially expressed genes that were significant at a p<0.05 level were subjected to Ingenuity Pathway Analysis (IPA). The top GO (Gene Ontology) terms for up and down-regulated genes are shown in Table 1. However, only up-regulated genes involved in response to unfolded protein, response to protein stimulus, protein folding and response to organic substance achieved genome-wide significance (a complete list, including the genes that contributed to each GO category, are shown in Table S3).

**Table 1:** GO (Gene Ontology) Analysis of Differentially Expressed Genes.

Top GO Terms: up-regulated genes	p-value	FDR
GO:0006986∼response to unfolded protein	1.01E-11	1.60E-08
GO:0051789∼response to protein stimulus	2.98E-11	4.71E-08
GO:0006457∼protein folding	4.82E-10	7.60E-07
GO:0010033∼response to organic substance	3.94E-06	0.006216
GO:0043066∼negative regulation of apoptosis	2.35E-04	0.369557
GO:0043069∼negative regulation of programmed cell death	2.60E-04	0.409938
GO:0060548∼negative regulation of cell death	2.66E-04	0.418438
GO:0034622∼cellular macromolecular complex assembly	5.69E-04	0.894464
GO:0006916∼anti-apoptosis	0.001366182	2.134406
GO:0009628∼response to abiotic stimulus	0.001462122	2.282681

Top GO terms: down-regulated genes	p-value	FDR
GO:0044271∼nitrogen compound biosynthetic process	0.001976523	2.898112
GO:0006397∼mRNA processing	0.009621474	13.3863
GO:0016071∼mRNA metabolic process	0.016933191	22.42004
GO:0006396∼RNA processing	0.023041571	29.28497
GO:0000375; GO0000398; GO0000377 RNA splicing	0.024247628	30.57157
GO:0007626∼locomotory behavior	0.025051429	31.41689
GO:0008380∼RNA splicing	0.028093909	34.53038
GO:0030182∼neuron differentiation	0.032160887	38.48665
GO:0007610∼behavior	0.041281949	46.563
GO:0009309∼amine biosynthetic process	0.042864753	47.85949

**Figure 4:**
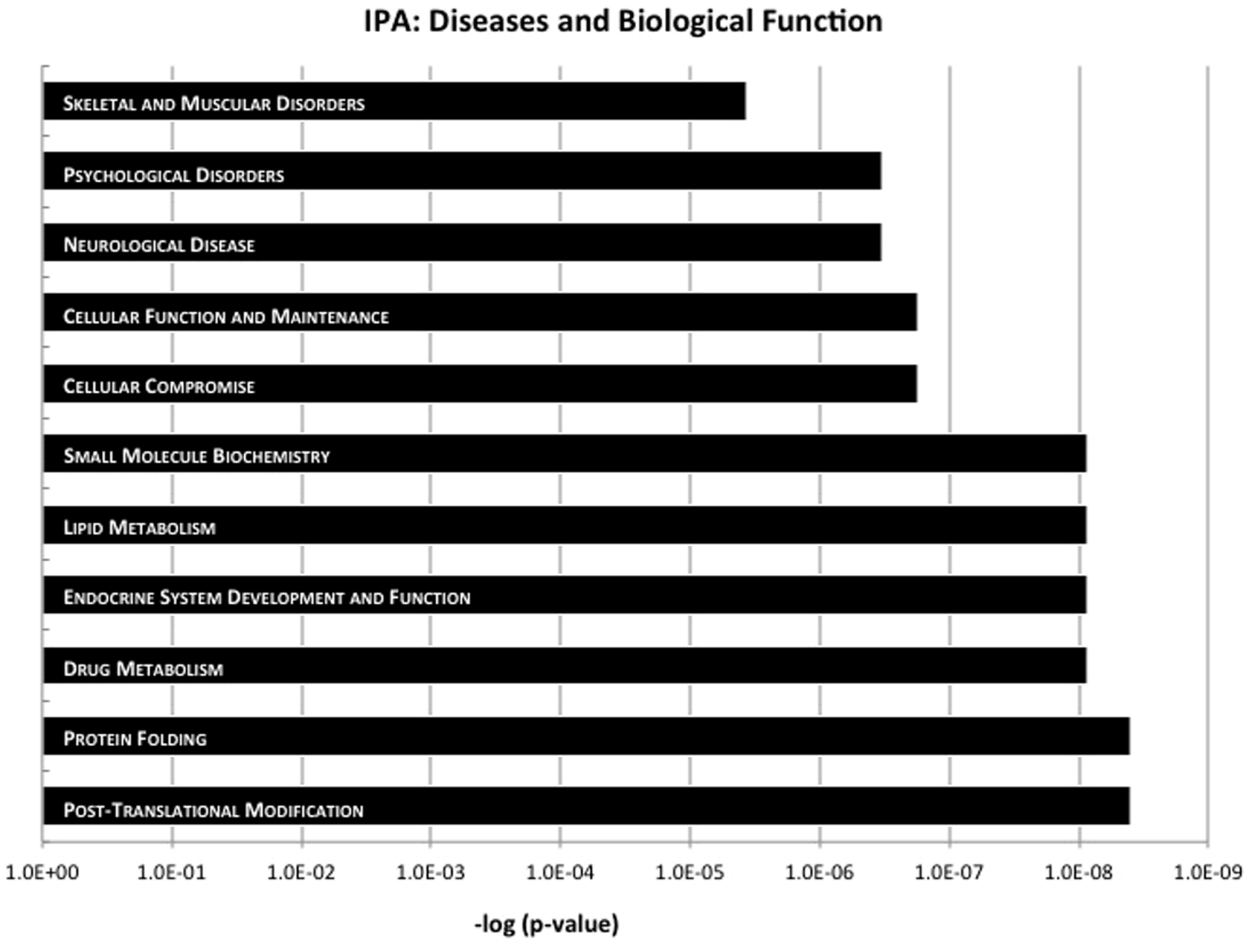
Ingenuity Pathway Analysis showing top diseases and biological functions of differentially expressed genes. All genes that were significant at a p<0.05 level were subjected to pathway analysis (Ingenuity Pathway Analysis; IPA). The top diseases and biological functions are shown.

The top diseases and biological functions from the IPA analysis are shown in Figure 4 (see Table S4 for entire set). This included, as expected, genes involved in protein folding. Interestingly, the top disease processes were neurological and psychological disorders, caused by differential expression of a number of genes implicated in SZ, BD and ASD, including *ZNF804A,* which decreased 1.7-fold, *SMARCA2*, which increased 1.7-fold, as well as *BAG3*, *KAT2A, HIST1H2BD, SLC6A8,* and *SLC17A7*, which were induced by HS, and *PRODH, ARNT2, DPP10, AHI1, IFITM1* and *RORA*, which decreased (described in detail in the discussion section).

The differentially expressed gene list was also examined using IPA's Upstream Regulator Analysis, which predicts factors that affect gene expression. As expected, genes responsive to HS factors (HSF) were the 1^st^ and 3^rd^ most significant upstream regulators (Table 2; Table S5). Interesting, the second most significant upstream regulator hit was RET, a member of the receptor tyrosine kinase family, which has well-established effects on cell growth and oncogenic transformation, as well the differentiation and survival of midbrain dopaminergic neurons [60]. *RET* CNVs have been found in a subgroup of patients with SZ [11].

**Table 2:** Upstream Regulator Analysis.

Up. Regulator	Mol. Type	z-score	p-value	Target molecules in dataset
HSF1	TR	0.875	5.50E-14	BAG3, CCT3, CLU, CRYAB, DNAJB1, EIF4A2, HMOX1, HSP90AA1, HSP90AB1, HSPA1A/HSPA1B, HSPA4L, HSPB1, HSPD1, HSPH1, KNTC1, SERPINH1, SLC5A3
RET	kinase	2.778	3.08E-09	CALB1, CLU, DNAJB2, FKBP4, FOS, HSP90AA1, HSPA1A/HSPA1B, HSPD1, HSPH1, STIP1, TH
HSF2	TR		3.02E-06	CCT3, CLU, HSPA1A/HSPA1B, HSPB1, HSPH1
HTT	TR		4.57E-06	CLK3, CRYAB, DNAJB1, FDFT1, FKBP4, FOS, HSP90AB1, HSPA1A/HSPA1B, HSPD1, KAT2A, MEIS2, MT-CO3, NGFR, NR4A1, PROM1, PURB, SERPINH1, TH, TUBA4A, UQCR11
CD437	CD	0	1.53E-05	APC2, CCT3, EIF3D, EIF4A2, FOS, HSP90AA1, NR4A1, PURB, RBMX, SNRPB
MMTP	CT	0.749	2.47E-05	CALB1, CASP9, FOS, HSPB1, NR4A1, TH
β-estradiol	CEM	−0.14	2.66E-05	ADCY1, ARNT2, BTG2, BUB3, CALB1, CALCA, CASP9, CLU, EIF3D, FDFT1, FGD6, FOS, HMOX1, HNRNPD, HSP90AB1, HSPA1A/HSPA1B, HSPB1, HSPD1, HSPH1, IER2, IFT122, MEIS2, NPR3, NR4A1, OGN, PROM1, SCG2, SETD7, SLA, STIP1, TH
NGF	GF	1.703	2.96E-05	BTG2, CALCA, ECEL1, FOS, HMOX1, NGFR, NR4A1, SCG2, TH
PDE	group		5.63E-05	FOS, HMOX1, NR4A1
KCl	CD	0.571	9.25E-05	BTG2, CALB1, FOS, NR4A1, SLC8A3, TH
liquiritigenin	CENM		1.09E-04	EPHX1, FOS, HMOX1
bisphenol A	CENM	1.941	1.86E-04	ARNT2, CLU, FOS, HSP90AB1, HSPA1A/HSPA1B, NR4A1
phencyclidine	CD		1.87E-04	DDC, FOS, SCG2

Abbreviations: Up. Regulator (Upstream Regulator); Mol. Type (Molecular Type P); TR (transcription regulator); z-score (activation z-score); p-value (p-value of overlap); GF (growth factor); MPTP (1-methyl-4-phenyl-1,2,3,6-tetrahydropyridine); CT (chemical toxicant); CD (chemical drug); CEM (chemical - endogenous mammalian); CENM (chemical - endogenous non-mammalian); KCl (potassium chloride)

Upstream Regulator Analysis using IPA predicts factors that affect gene expression.

Another upstream regulator connected to dopaminergic transmission in this analysis is MPTP, a neurotoxin that induces rapid nigrostriatal dopamine neuron degeneration, which is used to generate animal models of Parkinson Disease and can cause the disorder in humans [61]. Several other upstream regulator predictors are relevant to neuropsychiatric disorders, including genes whose expression patterns are affected by phencyclidine (PCP), a drug that mimics the symptoms of SZ in humans and in animal models, and bisphenol A, an endocrine disruptor used in the manufacture of polycarbonate plastics that has been implicated in ASD, and which down-regulates the ASD candidate gene *ARNT2* [8], [62]–[65]. Also among the top upstream regulators were CD437 (6-[3-(1-adamantyl)-4-hydroxyphenyl]-2-naphthalene carboxylic acid), a novel synthetic retinoic acid derivative, and NGF.

### lncRNAs

An emerging area of interest is the participation of long non-coding RNAs (lncRNAs) in the cellular response to HS and other cellular stresses [66], [67]. Three lncRNAs were affected by HS; RP11-386G11.10 and RP11-627G23.1, which decreased in expression, and RP11-50D9.3, which increased (Table S2). RP11-386G11.10 overlaps and is antisense to the tubulin A encoding genes *TUBA1A* and *TUBA1B*. RP11-627G23.1 is ∼25 Kb 3′ to *B3GAT1* (beta-1,3-glucuronyltransferase 1), which is highly expressed in the brain and is a key enzyme involved in the biosynthesis of the carbohydrate epitope HNK-1 that is present on a number of cell adhesion molecules important in neurodevelopment and hippocampal long-term potentiation [68]. *B3GAT1* is near a balanced translocation that segregates in a family with psychosis and depression, and an association to *B3GAT*2 has been found in SZ [68], [69]. Finally, RP11-50D9.3 is an antisense transcript that is ∼10 Kb 3′ to the HSP gene, *HSPA4L*, which is significantly induced in our study. Whether the induction of RP11-50D9.3 by HS is simply a byproduct of *HSPA4L* transcriptional activation, or is involved in regulating other genes involved in the stress response remains to be determined.

## Discussion

MIA can be viewed from two broad perspectives, which may have independent and overlapping effects on neurogenesis through the direct effects of immune cytokines that bind to their specific receptors on neurons, and by fever, which activates cellular stress pathways. In addition, it is likely that the response to MIA is influenced by genetic background; both fetal and maternal. So far, most biologically relevant studies have been carried out in animal models, with studies in humans restricted, with a few exceptions, to retrospective epidemiological studies. However, with the recent advent of iPSC technology there is now an opportunity to study some of the molecular consequences of MIA *in vitro* using human neurons. The technology also offers the opportunity to study patient-specific neurons to assess potential gene by environment (G × E) interactions.

In this study, we applied iPSC technology to study the effects of HS using a differentiation protocol that generates neuronal aggregates with characteristics of a developing first trimester telencephalon, a period of gestation that has been implicated in both SZ and ASD in some studies [38], [70]–[72]. A relatively brief exposure to HS resulted in a burst of gene expression changes, most notably in members of the HS family, including HSP70 and HSP90, and HSP binding partners. HSPs are ATP-dependent molecular chaperones that play a critical role in maintaining cellular homeostasis following HS and other stressors, such as nutritional deficiency, hypoxia, toxins, heavy metals, infections and inflammation; factors that have each been implicated as risk factors for SZ and ASD [73]–[82]. HSPs target misfolded proteins that accumulate in response to cellular stress, facilitating protein refolding and targeting damaged proteins for degradation in proteasomes. Neurodegenerative disorders, such as Alzheimer Disease, Parkinson Disease, and Huntington Disease, are caused by misfolded proteins, and activation of HSPs is being tested as a novel therapeutic strategy [83]–[86]. The aggregates used in these experiments and perhaps other neuronal induction methods derived from iPSCs, would be ideal systems to test the effects of a host of cellular stressors and the therapeutic effect of drugs on human neurons.

In addition to cellular stress, HSPs also play a role in the response to behavioral stress. HSP70 and HSP90, for example, act as glucocorticoid receptor chaperones aiding in their transport to the nucleus [87]–[89]. The adverse effects of chronic behavioral stress mediated in part by glucocorticoid-inducible genes, play an important role in depression and psychotic disorders [90]–[94]. Thus, the activation of HSP70 and HSP90 gene expression in response to HS and other cellular stressors may overlap with the brain's response to behavioral stress.

In addition to the marked induction of HS related genes - an expected finding - there were a number of genes of interest with respect to neurodevelopmental and neuropsychiatric disorders that showed substantial differences in expression, most notably *NGFR*, which exhibited the second most significant increase. Although NGF, the ligand for NGFR, was first discovered as a peripheral nervous system growth factor, it does have effects in the brain as well, especially as a trophic factor for cholinergic neurons [95]–[98]. NGF enhances neurite outgrowth in PC12 cells exposed to HS, a response that is affected by the atypical antipsychotic aripiprazole and *HSP90α* expression [99], [100]. It should be noted that NGF is also induced by IL-1, suggesting an overlap between the effects of HS and cytokines on NGF signaling [101].

As for a potential role for NGF signaling in neuropsychiatric disorders, a quantitative trait locus (QTL) in an *NGF* intron was recently found to be associated with nonverbal communication in ASD subjects [102]. Also, NGF levels are reduced in an animal model of Rett Syndrome and in the serum of medication-naïve patients with SZ [103], [104]. In addition, NGF-induced neurite extension is enhanced by DISC1 [105], [106].

In addition to *NGFR*, the expression of a number of SZ, BD and ASD candidate was significantly affected by HS, including *SMARCA2*, *HIST1H2BD, DPP10*, *SLC6A8, SLC17A7, ARNT2, AHI1* and *ZNF804A. SMARCA2,* which increased 1.7 fold with HS, encodes a REST-regulated, SWI/SNF chromatin-remodeling complex that has been implicated in SZ in a low density GWAS and CNV screening, and by molecular analysis following *REST* knockdown [46], [107], [108]. Point mutations in the gene have also been found in patients with Coffin–Siris syndrome and Nicolaides–Baraitser syndrome, which are characterized by severe developmental delay [109], [110]. Although we show a robust induction of *SMARCA2* expression, disease-associated *SMARCA2* mutations are primarily loss of function variants. However, it is well-established in psychiatric genetics that both copy gain and copy loss affecting the same locus can cause neurodevelopmental problems (15q11.2, 16p13.1 and 22q11.2, for example) [9], [111]–[113]. As for *HIST1H2BD*, it was one of 5 differentially expressed genes coding for histone variants found in a large study using lymphoblastoid cell lines derived from patients with SZ and controls [114]. Besides *HIST1H2BD*, there were 3 other histone variants (*HIST1H2BN, HIST1H1C* and *HIST1H2AC*) induced by HS that map to the same region on chromosome 6, near a GWAS signal in SZ, within a cluster of histone variants [115]. And, *DPP10*, *SLC6A8, SLC17A7*, *ARNT2*, and *AHI1* have been implicated in SZ, BD and ASD in GWAS, CNV analyses, exome sequencing and molecular studies [8], [10], [116]–[122].

Finally, the effect of HS on *ZNF804A* requires some discussion. The gene codes for a Zn-finger transcription factor that has been implicated in SZ and BD in replicated GWAS studies and molecular analysis [123]-[125]. In addition, rare copy gain and copy loss CNVs affecting the gene have been found in ASD, psychosis and anxiety disorder [10], [126]–[128]. A significant decrease in *ZNF804A* expression was found when C1 and C2 were exposed to HS, but an increase was detected in C3. Whether this variability is due to genetic variation within the gene and how this might relate to the GWAS findings remains to be determined; a much larger sample size will be needed.

Another gene implicated in SZ on a molecular level that was significantly affected by HS was *BAG3*, which codes for an HSP70 co-chaperone that mediates adaptive responses to stressful stimuli [75], [129], [130]. It has been found to be differentially expressed in the prefrontal cortex of patients with SZ, and in neurons derived from SZ-specific iPSCs [131], [132]. In addition to *BAG3*, SZ-specific neurons reported by Brennand et al. showed differential expression of 12 other genes that were also affected by HS in our experiment: *HSPA1A, HSPA1B, KAT2A, SAMD4A, PTPRE, UNC5C, GAS2L3, FGD6, CENPO, NALCN, ECEL1*, and *TM7SF2* [131]. The finding that genes involved in the cellular stress response are differentially expressed in SZ-specific neurons and in the brains of patients supports a role for these pathways in disease pathogenesis. Interestingly, *HSPA1B* was one of top four candidate genes, along with *DISC1, TCF4*, and *MBP*, identified in a comprehensive functional genomics analysis that combined genetic and molecular findings in SZ [2].

Other differentially expressed genes of interest in our HS experiment with respect to neuropsychiatric disorders were *PRODH* (proline dehyrdrogenase), *IFITM1* (Interferon Induced Transmembrane Protein), and several genes involved in dopamine transmission. *PRODH* maps to the 22q11.2 region deleted in velocardiofacial syndrome (VCFS), a haploinsufficiency disorder that leads to a variety of physical and psychiatric problems, including SZ and ASD, and *IFITM1* has been found to be differentially expressed in the brains of patients with SZ and ASD [133]–[141]. In addition, another member of the interferon-inducible family, *IFITM3*, along with the HS genes *HSPA6, HSPB8 and SERPINH1*, has been found to be differentially expressed in the brains of patients with ASD [29], [75].

Among the genes involved in dopaminergic function affected by HS were *DDC, PHOX2A, TH*, *DBH* and *ADCY1*. In addition, two potassium channel encoding genes, *KCNH1* and *KCND1*, increased with HS, while the ASD and BD candidate gene *DPP10* decreased; *DPP10* codes for a dipeptidyl peptidase that regulates potassium channel function [116], [117], [142]. Potassium channel function, which can affect dopaminergic tone, caused by a variety of genetic, autoimmune and molecular phenomena, is increasingly being recognized as a mechanism underlying the development of SZ, BD and ASD in subgroups of patients [143]–[147].

It is also important to note that in this study we identified genes that were differentially expressed in response to HS by combining the male and female samples. However, an analysis of gender differences in response to HS and other cellular stressors would be of great interest considering the 4-fold higher prevalence of ASD in males compared to females. Addressing this important issue, though, will require a larger sample size.

In summary, the findings reported here show that HS can induce changes in the expression of a number of candidate genes and pathways implicated in SZ, BD and ASD. In fact, the changes in expression that occur for some of these genes is equivalent to the effects of a CNV; ∼2-fold decrease as in a copy loss (*DPP10*, and *ARNT2* for example) or 50% increases and more, as in copy gains (*SMARCA2, NGFR*). The duration of the HS effect on neurogenesis, however, is likely to be transient, but that remains to be tested. Long term effects on gene expression could potentially occur as a result of the altered expression of chromatin regulators, such as *SMARCA2*, and *KAT2A*, and HS-inducible histone variants. However, even if the effects of HS are relatively brief, there may be subsets of neurons and neural progenitor cells that are severely impacted, especially in the context of other cellular stressors and genetic risk variants that can induce effects on gene expression that overlap with those affected by HS. Indeed, some cellular stressors known to operate through, or be influenced by HS pathways can be chronic, such as inflammation, exposure to heavy metals, and endocrine disruptors, as well as emotional stress; these might have a more protracted effect on gene expression and neurogenesis, truly mimicking the effects of CNVs; quantitatively and temporally.

Finally, in addition to the notion that a common environmental stressor like HS can have an effect on the expression of SZ, BD and ASD candidate genes similar in amplitude, if not duration, to a CNV, another important concept emerges from this study. That is, the role that some candidate genes have on disease pathogenesis may be underestimated if based solely on genetic findings. Although genes like *SMARCA2* and *ARNT2* might be viewed as relatively minor factors based on the small number of positive molecular and genetic studies that have been reported so far, their altered expression in response to HS suggests that they, and similarly affected candidate genes, could play a much more substantial role in SZ and ASD - as targets of common environmental stressors.

## Supporting Information

File S1 Text S1. Supplementary methods
Table S1. RNA-seq statistics. C1 and C2 refer to controls 1 and 2: HS1 and HS2 are the heat shocked counterparts.
Table S2. RNA-seq reads in TPM (Transcripts Per Million) for all genes arranged by p-value (lowest to highest). The 186 genes that showed nominally significant differences in the mean log2 fold-change (HS/control) are in bold type.
Table S3: Top Gene Ontology (GO) Terms for genes up and down-regulated by HS.
Table S4. Ingenuity Pathway Analysis showing disease and biological functions of differentially expressed genes
Table S5: Upstream Regulator Analysis, all genes
(ZIP)
