# Supplementary material for: Heat Shock Alters the Expression of Schizophrenia and Autism Candidate Genes in an Induced Pluripotent Stem Cell Model of the Human Telencephalon
Source: PLoS One. 2014 Apr 15;9(4):e94968. doi: 10.1371/journal.pone.0094968 (PMC3988108; doi:10.1371/journal.pone.0094968)
Supplement: File S1 — Text S1. Supplementary methods Table S1. RNA-seq statistics. C1 and C2 refer to controls 1 and 2: HS1 and HS2 are the heat shocked counterparts. Table S2. RNA-seq reads in TPM (Transcripts Per Million) for all genes arranged by p-value (lowest to highest). The 186 genes that showed nominally significant differences in the mean log2 fold-change (HS/control) are in bold type. Table S3: Top Gene Ontology (GO) Terms for genes up and down-regulated by HS. Table S4. Ingenuity Pathway Analysis showing disease and biological functions of differentially expressed genes Table S5: Upstream Regulator Analysis, all genes (ZIP) [file pone.0094968.s001.zip › test S1supplemental methods.docx]

**Supplemental Methods**

**Harvesting Fibroblasts**

Skin biopsies were performed in consenting individuals by a board-certified dermatologist. Skin biopsy samples were transferred to a small Petri dish containing 2-3 ml Skin Fibroblast Media (SFM) consisting of RPMI 1640, 10% FBS, 1% pen/strep, 10ng/ml FGF2. The sample was incubated at room temperature for 15 minutes. Medium was carefully aspirated and replaced with 1-2 ml of collagenase type II solution (3mg/ml collagenase II dissolved in DMEM High Glucose [Worthington Biochemical Corp. Lakewood, NJ: GIBCO/Invitrogen, Carlsbad, CA]). The tissue was chopped into small pieces using 2 sterile scalpels, after which they were allowed to incubate at 37oC for 1­2 hours depending on size. The sample was then collected in a 15ml falcon tube and washed with SFM (serum free medium). Tissue was collected by centrifugation at 1200 rpm for 4 minutes. The pelleted sample was then suspended in SFM and plated in a T12.5 ml flask at 37oC in 5% CO2 for 3 days without changing medium or manipulation, to allow fibroblasts to adhere. Then cells were subsequently fed every 2 days with RPMI 1640 containing 10% FBS until a confluent culture was obtained (~3 weeks). The cells were reprogrammed into iPSCs as described in the main methods section of the paper.

**Establishing Human iPSCs**

iPSC reprogramming was carried out by nucleofection. One vial of cells was thawed out and placed in a T75 flask in DMEM/F12 supplemented with 10% FBS and fed every 2 days. Cells were grown to ~50% confluence (~4-5 days), after which they were trypsinized and subjected to nucleofection (~6 x105 cells). Reprogramming was carried out using an Amaxa 4D-Nucleofector (P2 Primary Cell Kit from Lonza cat# V4XP-2012, Program FF-135) with non-integrating plasmids containing OCT4, SOX2, KLF4, L-MYC, LIN28, and a p53 shRNA vector (Addgene Cat. # 27077, 27078, 27080), according to Okita et al., with some modifications (1-3). iPSCs were maintained on Matrigel plates in mTeSR1 medium (Stem Cell Technologies) with daily feeding in 37oC/5% CO2/85% humidity. .

**Germ line markers, establishing pluripotency by *in vitro* differentiation and karyotype**

Pluripotency for all iPSC lines was confirmed by immunocytochemistry using antibodies (Ab) against Tra-1-60, Tra-1-81, SSEA3 and SSEA4, which are expressed in pluripotent stem cells (not shown). In addition, the capacity to differentiate into all 3 germ layers was established by *in vitro* assays, as previously described (2, 4). The markers desmin (mesoderm), α-fetoprotein (endoderm), and βIII-tubulin (ectoderm) were used {{2227 (5-8). A list of the Ab used in the study is shown in Supplemental methods. Karyotyping was carried out by Cell Line Genetics (Madison WI). All lines had normal karyotypes**.**

**Neuronal differentiation**

iPSC colonies were maintained on matrigel in mTeSR™1 and differentiated cells were removed manually. The colonies were pretreated for 1 hour with the ROCK inhibitor Y27632 (50µM) in mTeSR™1, rinsed with DMEM/F12 and then dissociated with accutase for 10min at 37^o^C. The cells were rinsed with DMEM/F12 and collected. Approximately 3.0 x 10^6^ cells were aliquoted onto an AggreWell™ plate (Stemcell Technologies) containing 300 microwells, which leads to the formation of uniform aggregates containing ~10,000 cells each. These were cultured for 6 days in mTeSR™1 medium supplemented with 500ng/ml DKK-1, 1.5µg/ml BMPRIA-Fc and 10µM SB431542. On day 6, aggregates were removed from the AggreWell™ plate according to the Stem Cell Technology protocol, and transferred to a 24-well ultra-low attachment plate. On day 18, 1% N2 supplement was added to medium. On day 25, aggregates were plated onto a 4-well chamber slide coated with 10µg/ml PORN, 2.5µg/ml laminin and 50µg/ml fibronectin, and cultured in Neurobasal medium supplemented with 2% B27 and 2mM L-glutamine for an additional 25 days, after which the aggregates were detached manually for immunocytochemistry. For immunocytochemistry, aggregates were prepared by fixing with 4% paraformaldehyde and embedded in OCT, and sectioned (10µm) on a cryostat.

**Reverse transcribed PCR (RT-PCR) and quantitative real-time PCR (qPCR)**

Total RNA was extracted using a miRNeasy Kit according to the manufacturer’s instructions (Qiagen). An additional treatment with DNase1 (Qiagen, Valencia, CA) was included to remove genomic DNA. Reverse transcribed PCR (RT-PCR) was performed using a OneStep RT-PCR Kit (Qiagen, Valencia, CA) according to the manufacturer’s instructions. The cDNA was used as a template for quantitative PCR (qPCR), which was carried out using the ABI 7900HT Real-Time PCR System (Applied Biosystems, Foster City, CA). Each reaction consisted of cDNA, primers, and SYBR Green PCR Master Mix (Applied Biosystems, Foster City, CA) in an 8 μl volume. Melting curve analysis of target sequences showed that all primers used in this study generated amplicons that had a single peak, without primer-dimer artifacts. Primer concentrations were optimized prior to use in qPCR experiments. Relative changes in gene expression were calculated using the 2^-∆∆Ct^ method with β2-microglobulin (β2M) as a reference gene. Each qPCR was carried out in triplicate, with each triplicate data point repeated 3 times. For the triplicates, only samples that differed by <0.3 Ct values were used in the final calculations. Less than ~5% of samples fell out of this range. In addition, standard curves were generated for each gene using a 50-fold dilution range. qPCR experiments were only used in the final analysis if the slope of the Ct vs input curve was at least -3.0 and the correlation coefficient for triplicate sames was >0.98. Relative changes in gene expression were calculated using the 2^-∆∆Ct^ method with β2-microglobulin (β2M) or beta-actin as reference genes. Significant differences in gene expression were assessed using a two-tailed student T-test.

**Primers used in this study:**

**Gene Forward Reverse**

β2M GCTCGCGCTACTCTCTCTTT CAATGTCGGATGGATGAAAC

β-actin TCACCACCACGGCCGAGCG TCTCCTTCTGCATCCTGTCG

TBX1 CACTACCACCCGGACTCG GCATGGAATTCAGAATAATGTGG

OCT4 plasmid CATTCAAACTGAGGTAAGGG TAGCGTAAAAGGAGCAACATAG

KLF4 plasmid CCACCTCGCCTTACACATGAAGA GCGTAAAAGGAGCAACATAG

SOX2 plasmid TTCACATGTCCCAGCACTACCAGA TTGTTTGACAGGAGCGACGAT

L-MYC plasmid GGCTGAGAAGAGGATGGCTAC TTTGTTTGACAGGAGCGACGAT

LIN28 plasmid AGCCATATGGTAGCCTCATGTCCGC TAGCGTAAAAGGAGCAACATAG

HSP90AA1 AGGGGGAAAGGGGAGTATCT AAGACCATGTCAACCCTTGG

HSP90AB1 CCCCTGCTGGTGTCTAGTGT CACAACATCCAATCCTGCTG

ZNF804A CCAGCTCTCACCAGAACCTC GGTTGCAAAGGGATGACAGT

PRODH GCCTTATCGCTCGGCTCT CAGCAGGAGCAGTAAGCTCAG

HIST1H2BD AAGGCCGTCACCAAGTACAC AGCAAACCAGGATGAGTTGG

NGFR GTGGGACAGAGTCTGGGTGT GGTTCCATCTCAGCTCAAGG

HSPB1 CGTTCCTCCCAAAACTCTGA CTGCAGGCTGGTAGGGATTA

ARNT2              CCCATGAAAGTTCAGCCAAT CCATGCATCCATCAGAAGTG

SMARCA2 GGCTTCTTTTGTCACCCTGA CACCAACACCACATTCTTCG

AHi1                   ACCTGAAACCGGAAACTCCT TCCCTCATTTGCCTTCTCAC

**Antibodies used in this study:**

| **Antibody** | **Company** | **Catalog #** |
| --- | --- | --- |
| Anti-human Tra 1-60 | eBioscience | 12-8863-80 |
| Anti-human Tra 1-81 | eBioscience | 12-8883-80 |
| AF488 Anti-mouse/human SSEA-3 | eBioscience | 53-8833-71 |
| AF488 Mouse anti SSEA-4 | BD Pharmingen | 560308 |
| Anti-Tubulin, beta III isoform | Millipore | MAB1637 |
| Desmin Ab-1 | ThermoScientific | MS-376-S |
| Anti-human/mouseα-Fetoprotein | R & D | MAB1368 |
| PSD95 (mouse) | UC Davis/NIH NeuroMab Facility | 75-028 |
| Synaptophysin(rabbit) | Abcam | ab8049 |
| Anti-GAD65/67 | Sigma | G5163 |
| Ms anti- Vglut2 | Millipore | MAB5504 |
| Rabbit neuronal class III β-tubulin | Fisher | NC9168644 |
| Tbr1 | Abcam | Ab31940 |
| Sheep anti-Tyrosine Hydroxylase | Pel-Freez | P60101 |

References

1. Okita K, Matsumura Y, Sato Y, Okada A, Morizane A, Okamoto S, et al (2011): A more efficient method to generate integration-free human iPS cells. *Nat Methods* 8:409-412.

2. Lin M, Hrabovsky A, Pedrosa E, Wang T, Zheng D, Lachman HM (2012): Allele-biased expression in differentiating human neurons: implications for neuropsychiatric disorders. *PLoS One* 7:e44017.

3. Chen J, Lin M, Foxe JJ, Pedrosa E, Hrabovsky A, Carroll R, et al (2013): Transcriptome Comparison of Human Neurons Generated Using Induced Pluripotent Stem Cells Derived from Dental Pulp and Skin Fibroblasts. *PLoS One* 8:e75682.

4. Pedrosa E, Sandler V, Shah A, Carroll R, Chang C, Rockowitz S, et al (2011): Development of Patient-Specific Neurons in Schizophrenia Using Induced Pluripotent Stem Cells. *J Neurogenet* .

5. Muenthaisong S, Ujhelly O, Polgar Z, Varga E, Ivics Z, Pirity MK, et al (2012): Generation of mouse induced pluripotent stem cells from different genetic backgrounds using Sleeping beauty transposon mediated gene transfer. *Exp Cell Res* 318:2482-2489.

6. Pal R, Mamidi MK, Das AK, Bhonde R (2012): Comparative analysis of cardiomyocyte differentiation from human embryonic stem cells under 3-D and 2-D culture conditions. *J Biosci Bioeng* .

7. Takahashi K, Tanabe K, Ohnuki M, Narita M, Ichisaka T, Tomoda K, et al (2007): Induction of pluripotent stem cells from adult human fibroblasts by defined factors. *Cell* 131:861-872.

8. Takahashi K, Yamanaka S (2006): Induction of pluripotent stem cells from mouse embryonic and adult fibroblast cultures by defined factors. *Cell* 126:663-676.
